# Awareness and perception of physicians about forgery and counterfeiting in the medical field in Egypt

**DOI:** 10.1038/s41598-025-93729-4

**Published:** 2025-04-19

**Authors:** Ahmed Salah Elsayed, Marwa Ali Mwaheb, Asmaa Yonis Elsary, Khaled El Rashed, Amal R. Saleh

**Affiliations:** 1 Department of Forensic Medicine and Clinical Toxicology, Faculty of Medicine, Fayoum University, Fayoum Governorate, Egypt; 2 Department of Public Health and Community Medicine, Faculty of Medicine, Fayoum University, Fayoum Governorate, Egypt; 3 Department of Forensic Medicine Authority, Ministry of Justice, Cairo, Egypt

**Keywords:** Forging signature, Medical field, Egypt, Medical research, Risk factors

## Abstract

Medical records may act as one of the legal pieces of evidence in the court. A complete and correct medical record contains a chronological health history of the patient, and it is one of the keys to resolving cases of alleged malpractice. The aim is to emphasize the importance of strict rules and training to ensure medical documents are handled properly and the prevalence of signature forgery in medical reports. This cross-sectional descriptive study was conducted with a sample of 300 randomly selected physicians from hospitals in Fayoum, Egypt, between 2024 and 2025. According to the opinions of 133 physicians (44.3%), forgery and counterfeiting are relatively common in the medical sector; 81 physicians (27%) believed it is common, and 9 physicians (3%) considered it very common, especially in medical reports. 186 (75.6%) were forged medical reports. 185 (61.7%) blamed it on inadequate supervision, while 162 (54%) said it was done to get money. This is followed by 160 doctors (53.3%) who cite respect for the senior and following his instructions if asked to signature in place. Importantly, the overwhelming support for enhanced oversight and education indicates a collective recognition of the necessity for systemic changes to combat forgery in the medical field.

## Introduction

Health is a fundamental right for all individuals and is the most important part of efforts to achieve prosperity in accordance with the vision of the state. Health care security as a human right: “Everyone has the right to live in physical and spiritual prosperity, to live and obtain a good and healthy environment, and to obtain health services”^1–3^.

Every medical and health worker who provides individual health services is obliged to make medical record data. Thus, hospitals and their components have an obligation to make documented records about patients called medical records in the delivery of health services. Medical records have a significant impact on the quality of service to patients. In addition, medical records can be a documentation tool regarding all events related to patients while in health facility services and make a communication medium between health workers in supporting the importance of health services today and in the future^4,5^.

Medical records include a variety of information, both recorded in writing and recorded relating to patient identity, history taking, physical examination, laboratory results, diagnoses, and all types of medical services and actions obtained by patients, both inpatient, outpatient, and emergency services. Medical records are documents that contain records regarding patient identity, examination, treatment, actions, and other services that have been provided to patients^6,18^.

Medical records may act as one of the legal pieces of evidence in the court. A complete and correct medical record contains a chronological health history of the patient, and it is one of the keys to resolving cases of alleged malpractice. Even in some cases, corruptness proposed the medical records as an alibi to avoid the investigation process. Truth proofing of a disease in a medical record patient is the subject of an examination of a court case in the court and is a tool to make sure a judge knows the truth in a dispute that is being handled by him. Doctors who falsify medical records can be subject to ethical and disciplinary sanction^7,8^.

The crime of falsification or document counterfeiting is a highly complex crime. This is due to the various legal aspects that it presents. At the base of this crime are also the forensic experts who urge as fundamental for the good prosecution of criminal law and criminal procedural law. The crime of falsification or counterfeit document, as its name indicates, typifies as illegality any modification of a document that may influence the legal traffic^9,10^.

A signature is a combined result of the cumulative effect of a number of factors such as muscular control, coordination, health, age, frequency of writing, temperature, etc. The signatures that are practiced illegally and are not genuine are termed as forged signatures^11,12^.

This is the first paper in Egypt and the world that aims to explore the prevalence of signature forgery in medical reports, assess the awareness levels among physicians regarding this issue, and propose measures to mitigate its occurrence.

This paper also explores how well doctors understand the legal consequences of forging signatures on medical reports. It highlights the risks of facing criminal charges, losing medical licenses, and violating ethical standards. The aim is to emphasize the importance of strict rules and training to ensure medical documents are handled properly.

## Materials and methods

### Study design and participants

This cross-sectional descriptive study was conducted with a sample of 300 randomly selected physicians from hospitals in Fayoum, Egypt, between 2024 and 2025.

### Data collection

We collected the questionnaire from experts in the Forensic Medicine Authority based on the cases that were submitted to them and conducted an interview with doctors. What are the most important problems that doctors face? Based on that, the work team agreed on the common questions that cover the idea of this research.

Data were gathered using a semi-structured questionnaire, initially developed in Arabic and then translated into English. To ensure the instrument’s effectiveness, a pilot study was performed to test its clarity, reliability, and validity. Feedback from the pilot study led to revisions, addressing issues related to ambiguous, irrelevant, or redundant questions.

### Questionnaire structure

The final questionnaire comprised several sections:

- **Demographic information**: Age, sex, specialty, and years of experience.
- **Perceptions and awareness**: Physicians’ perceptions and awareness regarding counterfeiting and signature forgery in medical reports.
- **Practices**: Current practices related to detecting and preventing counterfeiting and forgery.

The finalized questionnaire was created using Google Forms, an online survey tool selected for its user-friendliness and efficiency in data collection and management.

### Data collection procedure

The Google Form link was distributed via social media platforms, including Whats App and Facebook, to maximize outreach and ensure a broad audience of physicians. This approach facilitated widespread dissemination and participation across various professional networks.

Responses were collected over a six-month period and subsequently analyzed to draw conclusions about physicians’ perceptions, awareness, and practices regarding counterfeiting and signature forgery in medical reports.

### Data analysis

The statistical package of social science (SPSS) software, version 22 (SPSS Inc., Chicago, IL, USA), was used for data analysis. Simple descriptive analysis using percentages and numbers for the qualitative data. The Chi-Square test is used to compare two or more qualitative groups. P-values less than 0.05 were regarded as statistically significant.

### Ethical consideration

This study was approved by the Human Research Ethics Committee of the Faculty of Medicine, Fayoum University (ethics approval number: R620.). The study was in accordance with the ICH-GCP Guidelines and applicable local and institutional regulations and guidelines that govern the ethics committee’s operation. The study’s brief description and aim were mentioned at the beginning of the questionnaire. Informed consent was obtained from all study participants. The personal data of the respondent was kept private and confidential.

### Data availability

The database used and analyzed during the current study is available from the corresponding author on reasonable request.

## Results

Among the physicians included in the study, 163 (53.7%) were between the ages of 30 and 40 years old. 161 (53.7%) were males, and 139 (46.3%) were females. Internal medicine specialists made up 106 (35.3%) of the study sample, followed by ICU and anesthesia physicians, 62 (20.7%). In terms of years of experience, 100 (33.3%) of workers have worked for fewer than five years, while 98 (32.7%) have worked for six to ten years. As shown in Table 1.

**Table 1:** Description of demographic characters among study group.

Variables	Number (*n* = 300)	
	No.	%
Age (years)		
Less than 30 years	8	26.7%
Between 30–40 years	163	54.3%
Between 40–50 years	35	11.7%
More than 50 years	22	7.3%
Sex		
Male	161	53.7%
Female	139	46.3%
Speciality		
Emergency medicine	56	18.7%
ICU/anesthesia	62	20.7%
Internal medicine	106	35.3%
Surgical	38	12.7%
Forensic medicine and clinical toxicology	23	7.7%
General practitioner	15	5%
Experience duration		
Less than 5 years	100	33.3%
Between 6–10 years	98	32.7%
Between 11–20 years	77	25.7%
More than 20 years	25	8.3%

According to the opinions of 133 physicians (44.3%), forgery and counterfeiting are relatively common in the medical sector; 81 physicians (27%) believed it is common, and 9 physicians (3%) considered it very common, especially in medical reports. 186 (75.6%) were forged medical reports. 185 (61.7%) blamed it on inadequate supervision, while 162 (54%) said it was done to get money. In patient files or medical reports, 228 doctors (76%) cite the mutual trust among colleagues as the primary justification for having a doctor signature in place of a colleague. This is followed by 160 doctors (53.3%) who cite respect for the senior and following his instructions if asked to signature in place. As shown in Table 2.

**Table 2:** Prevalence, types and causes of forgery and counterfeiting from study physicians’ point of view.

Items	Frequency	
	No	%
Forgery and counterfeiting are common in the medical field		
Very rare	16	5.3%
Rare	61	20.3%
Moderate	133	44.3%
Common	81	27%
Very common	9	3%
Types of forgery and counterfeiting in medical filed (*n* = 246)		
Forged medical reports	186	75.6%
Counterfeit medical prescriptions	105	42.7%
Fake research and studies	91	36.9%
Fake medical licenses	53	21.5%
Forged certificates and academic qualifications	59	23.9%
Reasons lead to forgery and counterfeiting in the medical field		
Seeking financial profit	162	54%
Work stress	132	44%
Lack of supervision	185	61.7%
Lack of awareness and training	132	44%
Reasons that make a doctor sign instead of his colleague in patient files or medical reports		
Mutual trust between colleagues	228	76%
Respect the senior and carry out his directions if he is asked to sign instead	160	53.3%
Fear of oppression from his superiors at work if he refrained from carrying out their orders to sign in their place	81	27%
Good faith and ignorance of the laws and regulations	153	51%
Desire to complete tasks and not disrupt work	88	29.3%

According to the study’s physicians’ awareness of forgery and counterfeiting, 126 (42%) of them were aware that signing a patient’s file under a doctor’s name rather than the colleague responsible for the case is a kind of forgery. Of them, 197 (65.7%) were aware that writing a medical report and signing it under the name of a different physician than the one handling the case is seen as a kind of forgery. Because the physician signed the death report instead of his colleague, 237 (79%) of them were aware that they were legally responsible. Almost all of them, 286 (95.3%), were aware that if an issue arises from signing medical documents in place of a colleague, the good faith and trust in colleagues would not shield them from legal responsibility. As shown in Table 3.

**Table 3:** Awareness about forgery and counterfeiting among study physicians.

Items	Frequency	
	No	%
The signature of a doctor instead of his colleague on the patient’s file is a type of forgery		
Yes	126	42%
No	108	36%
Do not know	66	22%
Writing a medical report and signing it with the name of another doctor instead of the doctor who is responsible for the case considered a type of forgery		
Yes	197	65.7%
No	40	13.3%
Do not know	63	21%
In the event that one of your colleagues asked you to sign a death report instead of him, and then it became clear that there was a criminal suspicion in the death and the prosecution requested that the body be exhumed for autopsy… Do you think that you have legal responsibility as a result of your signature in place of your colleague		
Yes	237	79%
No	19	6.3%
Do not know	44	14.7%
Do you think that your good faith and trust in your colleagues exempt you from legal accountability in the event of a problem resulting from your signing instead of your colleague on medical documents?		
Yes	14	4.7%
No	286	95.3%

According to the current study, 246 (82%) of physicians had dealt with cases of forgery or counterfeiting in the medical field. However, 127 (42.3%) of physicians had signed a patient file or medical report in place of one of their colleagues; 61 (48%) of them had done so a few times, while 29 (22.9%) had done so frequently. Only 24 (8%) of them had a problem as a result of signing a medical paper instead of colleagues. As shown in Table 4.

**Table 4:** Practicing forgery and counterfeiting among study physicians.

Items	Frequency	
	No	%
Did you encountered cases of forgery or counterfeiting in medical field		
Yes	246	82%
No	54	18%
Did you ever signed a substitute for one of your colleagues on a patient file or medical report		
Yes	127	42.3%
No	124	41.3%
Do not remember	49	16.3%
How many times you signed a substitute for one of your colleagues (*n* = 127)		
Rare	28	22%
Few times	61	48%
Many times	29	22.9%
Always	9	7.1%
Did you encountered a problem as a result of your signature on a medical document in place of one of your colleagues or the signature of one of your colleagues in place of you		
Yes	24	8%
No	276	92%

Assessment of physician perception towards forgery and counterfeiting revealed that 141 (47%) of the study population agreed to signature medical reports instead of their colleagues. but 129 (43%) of physicians completely refuse to signature a report for a patient that they never examined, although 101 (33.7%) agreed to signature with their own name and the name of the colleague who asked to signature the report. 220 (73.3%) of physicians agreed to modify the treatment plan and signature the file with their names based on the instructions of the consultant. 118 (39.3%) of physicians agreed and accepted modifications to the treatment plan done by their colleagues and signed by the physician’s name, but at the same time, the physician insisted on being informed of these modifications before writing them in the medical file. On the other hand, 95 (31%) of physicians did not accept their colleagues modifications and elevated the complaint to their consultant. If the patient had modified the medical report, 140 (46.7%) of physicians would inform the hospital administration to deal with that patient, and 104 (34.7%) of physicians would take the report from the patient and dispose of it. If a nurse signed on behalf of a physician in the drug-dispensing notebook, 163 (54.3%) of physicians will inform the head nurse that it is illegal for a nurse to signature the drug- notebook instead of a doctor. In cases of a nurse signing on behalf of the physician in the drug dispensing notebook, 95 (31.7%) of physicians will review the dose, and if it is correct, he will signature it and warn the nurse not to repeat it. As shown in Table 5.

**Table 5:** Perception about forgery and counterfeiting among study physicians.

Items	Frequency	
	No	%
If one of your colleagues asked you to sign his name on medical reports and gave you his ID number to add to the signature- would you accept or refuse?		
I agree	141	47%
I refuse	159	53%
What would you do if one of your colleagues or superiors at work asked you to sign instead of him a medical report for a patient whom you had never examined?		
I agree to sign my name on the report	13	4.3%
I agree to sign the name of my colleague or superior who asked me to do so	57	19%
I agree to sign my name and write instead of (Dr/………) and write the name of my colleague or superior who asked me to do so	101	33.7%
I refuse to sign a medical report for a patient I have not examined before	129	43%
What would you do if a consultant asked you to modify a patient’s treatment plan and write that in his medical file		
I modify the treatment plan and sign the file with my name	21	7%
I modify the treatment plan and sign the file with the consultant’s name	59	19.7%
I modify the treatment plan, sign the file with my name, and write based on the instructions of the consultant (Dr./……….)	220	73.3%
While following up on a patient’s condition in the inpatient department, you were surprised that one of your colleagues modified the treatment plan in the medical file and signed your name… What would you do		
There is no problem if modifying the treatment plan is in the patient’s best interest	38	12.7%
I agree to the modification and talk to my colleague and tell him that I should have been informed of the modification before writing it in the medical file	118	39.3%
I refuse the modification and record it in the patient’s medical file and inform the nursing staff that I am the only one responsible for following up on my patients	49	16.3%
I informed my boss that one of my colleagues modified the treatment plan for one of my patients and signed my name without consulting me	95	31.7%
While following up on one of your patients, you discovered that he had modified the medical report to increase the length of leave granted to him…. What would you do		
I take the report from him and dispose of it	104	34.7%
I inform the hospital administration to deal with him	140	46.7%
I inform the police	19	6.3%
I ignore his conduct as long as he does not change the medical information in the report.	37	12.3%
While prescribing a dose of an opioid to a patient, a nurse signed on your behalf in the drug dispensing notebook and wrote your ID number because you were busy examining other cases…. What would you do		
Nothing… it’s normal as long as the drug dose is written correctly in the notebook.	16	5.3%
I review the written dose and if it is correct, I sign it and warn him not to repeat it	95	31.7%
I inform the head nurse that it is illegal for a nurse to sign the drug notebook instead of a doctor	163	54.3%
I raise the matter to the hospital administration to take the necessary legal measures	26	8.7%

More than 60% of physicians who participated in the study thought that forgery caused multiple consequences in the form of loss of confidentiality between patient and doctor, legal problems, and poor reputation of the medical institution. 251 (83.7%) of physicians thought there is a need to strengthen oversight and legislation to prevent forgery and counterfeiting in the medical field. To reduce forgery and counterfeiting in the medical field, 197 (65.7%) of physicians suggested strengthening supervision and inspection, and 142 (47.3%) of them suggested improving training and education. As shown in Table 6.

**Table 6:** Forgery consequences and suggestions for its prevention among study physicians.

Variables	Frequency	
	No	%
Effects of forgery in the medical field		
Negative impact on patients’ health	159	53%
Loss of confidence between patient & doctor	192	64%
Legal problems	186	62%
Poor reputation of the medical institution	191	63.7%
There is a need to strengthen oversight and legislation to prevent forgery and counterfeiting in the medical field		
Yes	251	83.7%
No	32	10.7%
Do not know	17	5.7%
Measures can help reduce forgery and counterfeiting in the medical field		
Strengthening supervision and inspection	197	65.7%
Increasing legal penalties	117	39%
Improving training and education	142	47.3%
Community awareness	134	44.7%

## Discussion

In medical practice, the fields of ethics, law, and professional standards are closely interlinked. Ethics, often described as the science of customs or the study of moral principles, guides what is considered appropriate behavior within the profession. When ethical standards are violated, the repercussions differ from those of legal violations. Ethical breaches do not typically invoke formal legal penalties. Instead, sanctions for ethical misconduct are determined and enforced by the professional community itself. This group, which establishes and upholds the ethical code, imposes these sanctions based on the agreed-upon standards of the profession^9,13,14^.

In this recorded study, 53.7% of physicians were aged between 30 and 40 years. The sample comprised 53.7% males and 46.3% females. Internal medicine specialists constituted 35.3% of participants, followed by ICU and anesthesia physicians at 20.7%. Regarding experience, 33.3% of physicians had less than five years of experience, while 32.7% had six to ten years of experience. According to the study, 44.3% of physicians believe forgery and counterfeiting are relatively common in the medical sector, with 27% considering it common and 3% very common, particularly in medical reports, as 75.6% of physicians who acknowledged this issue. 61.7% of physicians attributed it to inadequate supervision, while 54% cited financial motives. Regarding the practice of signing on behalf of colleagues, 76% of doctors identified mutual trust among colleagues as the primary reason, followed by 53.3% of doctors who cited respect for senior colleagues and compliance with their instructions.

Despite this awareness, the current study also reveals a complex landscape of acceptance and practice among physicians. While many acknowledge the ethical breaches involved, a substantial number still engage in signing documents for colleagues, driven primarily by mutual trust and respect for seniority. This duality illustrates the tension between ethical standards and practical realities in medical practice. Some individuals may resort to falsifying or fabricating official documents, particularly those that are meant to be taken seriously, such as driver’s licenses, bank checks, prescriptions, cash, or even court testimonies. This unlawful manipulation of genuine documents is often driven by the desire to gain personal advantages^12,15^.

This research also revealed that 42% of physicians recognized that signing a patient’s file under another doctor’s name constitutes forgery. Additionally, 65.7% of physicians acknowledged that drafting and signing a medical report under a different physician’s name is also considered forgery. Furthermore, 79% of physicians understood that signing a death report on behalf of a colleague carries legal responsibility. Almost all participants, 95.3%, were aware that legal responsibility cannot be avoided by relying on good faith and trust when signing medical documents in place of a colleague. The inadequate awareness of the physicians of current legislation in Egypt on anti-counterfeiting of medical reports, as this lack of knowledge may greatly affect their attitudes and practices^16^.

The study found that 82% of physicians had encountered cases of forgery or counterfeiting in the medical field. Among them, 42.3% had signed patient files or medical reports on behalf of colleagues. Of these, 48% had done so occasionally, while 22.9% had done so frequently. Only 8% of physicians reported facing problems as a result of signing medical documents in place of their colleagues.

The assessment of physician perceptions toward forgery and counterfeiting revealed the following:

Signing Reports shows 47% of physicians approved to sign medical reports on behalf of colleagues. Conversely, 43% of physicians refused to signature reports for patients they had not examined. Additionally, 33.7% of physicians were willing to signature reports using both their own name and the name of a colleague who requested it^17^. Modifications to treatment Plans documented 73.3% of physicians coincide to modify treatment plans and signature the files based on a consultant’s instructions. Meanwhile, 39.3% of physicians accepted modifications made by colleagues and signed the reports, provided they were informed of these changes beforehand. In contrast, 31% of physicians did not accept such modifications and reported them to their consultant. Handling modified medical reports if a patient modified a medical report, 46.7% of physicians would inform hospital administration, while 34.7% of physicians would take and dispose of the modified report^18^.

Nurse signing drug dispensing notebooks in cases where a nurse signed on behalf of a physician in the drug dispensing notebook, 54.3% of physicians would inform the head nurse of the illegality of such actions. Additionally, 31.7% of physicians would review and signature the dose if correct while warning the nurse not to repeat the action^18^.

Over 60% of physicians participating in the study believed that forgery results in significant consequences, including breaches of patient confidentiality, legal issues, and damage to the reputation of medical institutions. Additionally, 83.7% of physicians expressed the need for enhanced oversight and legislation to combat forgery and counterfeiting in the medical field. To address these issues, 65.7% of physicians advocated for strengthened supervision and inspection, while 47.3% of physicians recommended improvements in training and education. The absence of penalties for those responsible for managing medical records creates an opportunity for potential misuse, as there are no consequences for negligent parties. Additionally, inadequate harmonization in regulatory frameworks, poor coordination among regulations, and ineffective regulatory planning contribute to the creation of inadequate policies^5,11^.

## Conclusion

The study underscores a pervasive concern regarding forgery and counterfeiting within the medical field, revealing significant insights into physician perceptions and practices. The data indicates that a considerable number of physicians are aware of and have encountered forgery, with 82% reporting experiences with such practices. A noteworthy proportion of participants recognized that signing medical documents on behalf of colleagues and other related practices constitute forgery, highlighting a widespread understanding of the legal and ethical implications.

In summary, addressing these issues through strengthened regulations and training is essential for maintaining the integrity of medical practice and ensuring patient trust in healthcare professionals.

## Data Availability

The database used and analysed during the current study available from the corresponding author on reasonable request.
